# Phenolics and Flavonoids Compounds, Phenylanine Ammonia Lyase and Antioxidant Activity Responses to Elevated CO_2_ in *Labisia pumila* (Myrisinaceae) 

**DOI:** 10.3390/molecules17066331

**Published:** 2012-05-25

**Authors:** Hawa Z.E. Jaafar, Mohd Hafiz Ibrahim, Ehsan Karimi

**Affiliations:** Department of Crop Science, Faculty of Agriculture, University Putra Malaysia, Serdang 43400, Selangor, Malaysia

**Keywords:** CO_2_ enrichment, phenolics, flavonoids, phenylalanine ammonia lyase activity (PAL), free radical scavenging (DPPH), ferric reducing antioxidant potential (FRAP)

## Abstract

A split plot 3 × 3 experiment was designed to examine the impact of three concentrations of CO_2_ (400, 800 and 1,200 µmol·mol^−1^) on the phenolic and flavonoid compound profiles, phenylalanine ammonia lyase (PAL) and antioxidant activity in three varieties of *Labisia pumila* Benth. (var. *alata*, *pumila *and *lanceolata*) after 15 weeks of exposure. HPLC analysis revealed a strong influence of increased CO_2_ concentration on the modification of phenolic and flavonoid profiles, whose intensity depended on the interaction between CO_2_ levels and *L. pumila* varieties. Gallic acid and quercetin were the most abundant phenolics and flavonoids commonly present in all the varieties. With elevated CO_2_ (1,200 µmol·mol^−1^) exposure, gallic acid increased tremendously, especially in var. *alata* and *pumila *(101–111%), whilst a large quercetin increase was noted in var. *lanceolata* (260%), followed closely by *alata* (201%). Kaempferol, although detected under ambient CO_2_ conditions, was undetected in all varieties after exposure. Instead, caffeic acid was enhanced tremendously in var. *alata* (338~1,100%) and *pumila *(298~433%). Meanwhile, pyragallol and rutin were only seen in var. *alata* (810 µg·g^−1^ DW) and *pumila* (25 µg·g^−1^ DW), respectively, under ambient conditions; but the former compound went undetected in all varieties while rutin continued to increase by 262% after CO_2_ enrichment. Interestingly, naringenin that was present in all varieties under ambient conditions went undetected under enrichment, except for var. *pumila *where it was enhanced by 1,100%. PAL activity, DPPH and FRAP also increased with increasing CO_2_ levels implying the possible improvement of health-promoting quality of Malaysian *L. pumila* under high CO_2_ enrichment conditions.

## 1. Introduction

Carbon dioxide is regarded as one of the most limiting factors in photosynthesis. Currently, the CO_2_ concentration in ambient atmosphere is about 380 µmol·mol^−1^ and it is expected to increase by 600 µmol·mol^−1^ CO_2_ by end of 2050 [1]. The prospect of improving photosynthesis in crops through CO_2_ enrichment has interested plant scientists for many years [2]. Enriching plants with high levels of CO_2_ has been proven to increase plant growth, morphology, development, and yield of many crops, and this response is a function of CO_2_ rate and duration of exposure [3]. Crops under enriched CO_2_ atmosphere acquire positive features with enhanced plant adaptation and growth. The greatest advantages of CO_2_ enrichment is in the enhancement of leaf gas exchange capacity, particularly under undesirable climatic conditions [4].

Plant antioxidants have been a focus of attention in recent years due to the health preservation functions of these components that can help reduce the threat of chronic diseases such as cancer, diabetes and hypertension. This is attributed to the high scavenging activity of antioxidants towards free radicals that are usually associated with these diseases [5]. It is currently known that phenolic acids and flavonoids are antioxidants with high anti-inflammatory and anti-carcinogenic activities [6,7]. It is also known that phenolics and flavonoids can function as reducing agents, free radical scavengers and quenchers of singlet oxygen formation [8]. High contents of natural flavonoids and phenolic acids are found in green tea, fruits, and vegetables, while some amounts of phenolics also exist in red wine and coffee [9]. These components of polyphenols have been proven to have significant roles in the curing cancer and other human ailments [10]. The concentration of phenolics and flavonoids in plants varies among organs, tissues and developmental stage, and is influenced by environmental factors such as temperature, UV and visible radiation, nutrient and water availabilities, and atmospheric CO_2_ concentration [11].

The exposure of medicinal plants to high level of CO_2_ may give positive response in the form of increased anti-oxidative properties [12]. Furthermore, several researchers have found increase in anti-oxidative activity under elevated CO_2_ exposure due to increases in the levels of some secondary metabolite compounds that might up-regulate the antioxidant activity [13,14]. As it is generally known that secondary metabolism and primary metabolism are inter-related by the rates at which substrates are diverted from primary pathways and channeled into the secondary biosynthetic routes [15]. Several environmental factors affecting growth, photosynthesis and other parts of primary metabolism will also affect secondary metabolism [16]. Resource allocation hypotheses such as the carbon-nutrient balance (CNB) [17] and growth differentiation balance (GDB) [18] have been proposed by researchers to predict the effects of increased CO_2_ concentration on plant secondary metabolites. They assume that changes in carbon source-sink relationship, as a consequence of carbon availability, determines variations in the relative partitioning of carbon to growth and production of carbon based secondary metabolites (CBSM). Recently, in Malaysia, it has been found that the enrichment of *Labisia pumila* and *Zingiber officinale* with high levels of CO_2_ could increase the secondary metabolite production [19,20]. The same result was in agreement with data previously obtained from *Pinus taeda* [4], *Podophyllum hexandrum* [21], *Digitalis lanata* [22], *Brassica oleracea* [23] and *Hypericum perforatum* [24]. 

*Labisia pumila*, Benth. popularly known as Kacip Fatimah, is a sub-herbaceous plant with creeping stems from the family Myrsinaceae that is found widespread in Indochina and throughout the Malaysian forest. Recently, it was found that this herb is a good source of natural antioxidants due to its high content of anthocyanins, flavonoids, and phenolic acids [25,26]. Results of elevated CO_2_ imposition on total phenolics and flavonoids have been reported by Ibrahim *et al*. [27], however, there is no available information on the effects of CO_2_ concentration on the profiling of phenolics and flavonoids composition and antioxidant activity of this plant under high CO_2_ conditions, especially for three recognized varieties of this plant (var *alata*, *pumila* and *lanceolata*). Previous research has shown that there no significant differences in the total phenolics, flavonoids and carbohydrate content between those varieties [28]. This study was performed to evaluate the effects of elevated CO_2_ enrichment on leaf of phenolics and flavonoids compounds in the three varieties of *L. pumila*. The DPPH, FRAP activity and phenylalanine ammonia lyase (PAL) were also determined in the study. 

## 2. Results and Discussion

### 2.1. HPLC Analysis of Phenolics

In the study, initially, four phenolics compounds were determined, *i.e.*, (gallic acid, pyragallol, caffeic acid and salicylic acid). However, only three phenolics compounds were revealed to be present as no salicylic acid was detected in all three varieties under ambient levels or exposure to elevated CO_2_ conditions. 

**Figure 1:**
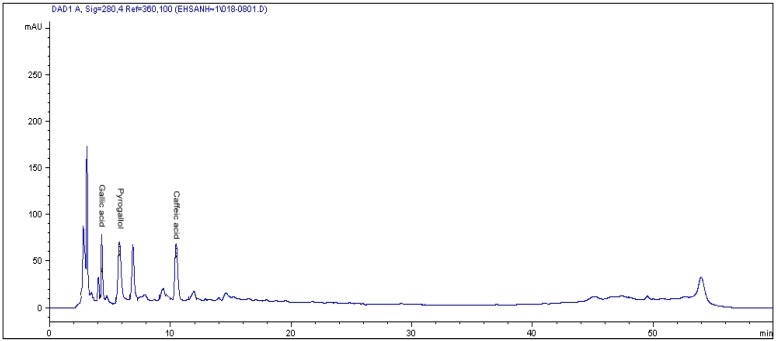
The RP-HPLC chromatogram of phenolics compounds in the leaves of *Labisia pumila*. Identification of compounds: gallic acid, pyrogallol and caffeic acid.

Leaf profiling results for phenolic compounds were observed to be significantly influenced by the interaction between CO_2_ and varieties (*p* ≤ 0.01; Table 1; Figure 1). Among the phenolic acid compounds profiled, gallic acid exhibited the highest concentration. With increasing CO_2_ the gallic acid levels increased tremendously, but at different rates for the different varieties under different CO_2_ concentrations. Increased CO_2_ levels from 400 to 800 µmol·mol^−1^ CO_2_, saw the gallic acid component in var. *alata* improve by 86% (448 µg·g^−1^ dry weight *vs*. 837 µg·g^−1^ dry weight) followed by var. *pumila* (30%) and *lanceolata* (16.4%) (Table 2). However further exposure to 1,200 µmol·mol^−1^ CO_2_ showed a remarkable improvement of gallic acid in var. *lanceolata* (130%), more than var. *alata* (111%) and var. *pumila* (101%), implying that increased CO_2_ levels had a direct impact on gallic acid concentration that varied among varieties. Under exposure to elevated CO_2_ (1,200 µmol·mol^−1^) the gallic acid content of var. *alata *and *lanceolata* was higher than pomegranate (901 µg·g^−1^ dry weight), but lower than onion that recorded 1,490 µg·g^−1^ dry weight. The present data indicate that enrichment of *L. pumila* with high CO_2_ can possibly enhance the production of gallic acid in the three varieties. 

**Table 1:** Phenolics content (µg·g^−1^ dry weight) in three varieties of *Labisia pumila *grown under different CO_2_ concentration.

CO_2_ levels (µmol·mol^−1^)	Varieties	Gallic acid *	Pyragallol	Caffeic acid
	***Alata***	448.12 ± 2.44 ^d^	810.03 ± 2.44	47.83 ± 3.22 ^f^
**400**	***Pumila***	215.48 ± 4.32 ^g^	ND	43.92 ± 2.11 ^f^
	***Lanceolata***	406.03 ± 3.22 ^f^	ND	115.21 ± 1.14 ^e^
	***Alata***	837.434 ± 0.87 ^b^	ND	215.51 ± 2.54 ^c^
**800**	***Pumila***	282.17 ± 0.43 ^g^	ND	177.35 ± 2.56 ^d^
	***Lanceolata***	474.33 ± 3.67 ^c^	ND	ND
	***Alata***	948.28 ± 6.77 ^a^	ND	543.88 ± 3.44 ^a^
**1200**	***Pumila***	435.69 ± 9.87 ^e^	ND	237.86 ± 5.66 ^b^
	***Lanceolata***	935.91 ± 4.34 ^a^	ND	ND

ND = not detected. All analyses are the mean of nine measurements ± standard error of mean. Results expressed in µg·g^−1^of dry plant material. Means not sharing a common letter were significantly different at *p* ≤ 0.05.*

**Table 2:** Percent of increase or decrease of phenolics compounds in three varieties of *Labisia pumila* grown under elevated CO_2_ concentration (800 and 1,200 µmol·mol^−1^).

Phenolic compounds	Var. *alata*		Var. *pumila*		Var. *lanceolata*	
	800 (µmol·mol^−1^)	1,200 (µmol·mol^−1^)	800 (µmol·mol^−1^)	1,200 (µmol·mol^−1^)	800 (µmol·mol^−1^)	1,200 (µmol·mol^−1^)
**Gallic acid**	+86%	+111%	+30%	+101%	+16.4%	+130%
**Pyragallol**	−100%	−100%	0%	0%	0%	0%
**Caffeic acid**	+338%	+1010%	+298%	+433%	−100%	−100%

Results expressed in percent; + and − indicate respectively increase and decrease of component concentrations when exposed to CO_2_.

What was more interesting with modification of CO_2_ is the non-detectability of pyragallol in var. *alata* when exposed to 800 and 1,200 µmol·mol^−1^ CO_2_ although the component were only detected in var. *alata* at high concentration under ambient CO_2_ condition. A similar effect was observed with var. *lanceolata* where caffeic acid was not detected when the plant exposed to elevated CO_2_ (800 and 1,200 µmol·mol^−1^ CO_2_), although a significant increase in caffeic acid concentration was observed in both var. *alata* and *pumila*. The caffeic acid concentration recorded increases by 338% and 1010% for var. *alata* and 298% and 433% for var. *pumila* at 800 and 1,200 µmol·mol^−1^ CO_2_ respectively. A similar result was obtained by Lindroth *et al*. [29] whereby exposure to 650 µmol·mol^−1^ CO_2_ had increased phenolic compounds (gallic acid, phenolic glycosides, gallotannin, and ellagitannin) in aspen, oak and maple seedlings. Vurro *et al.* [30] also saw increased phenolics components in *Thymus vulgaris* with exposure to 500 µmol·mol^−1^ CO_2_. They also found supplementation with elevated CO_2_ increased the superoxide dismutase and gluthatione reductase activity and essential content in the plants. The current results exhibited an enhanced of the medicinal properties of *L. pumila*, particularly the gallic acid compounds through CO_2_ enrichment. The increase in phenolics compounds in the current study under elevated CO_2_ might be attributed to increased *L. pumila* growth under CO_2_ exposure that enhanced the concentration of phenolics per gram dry mass basis of *L. pumila* [31]. The current result showed an agreement with CNB and GDB hypotheses [17,18] that predict an increase in phenolics content under elevated CO_2_ conditions. From previous study by Ibrahim and Jaafar [27] it was found that the total phenolics and flavonoids have a significant positive correlation with total biomass and specific leaf leaf area that indicated the increase in secondary metabolites content in *L. pumila* was due to enhanced plant morphology that might contain more plant secondary metabolites [19].

### 2.2. HPLC Analysis of Flavonoids

**Figure 2:**
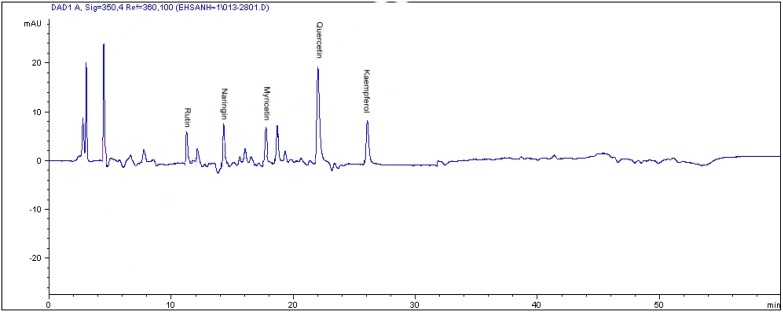
The RP-HPLC chromatogram of flavonoids compounds in the leaves of *Labisia pumila*. Compound identification as labeled.

The results obtained from the analysis of flavonoids are shown in Table 3. Five flavonoid compounds were analyzed, *i.e*., rutin, naringenin, myrecetin, quercetin and kaempferol (Figure 2). From the result, it observed that the production of kaempferol became undetectable in the three *L. pumila* varieties when the plants were exposed to elevated CO_2_. Under ambient CO_2_ conditions kaempferol content was found to be higher in var. *pumila* (221.91 µg·g^−1^ dry weight), followed by var. *alata* (186.71 µg·g^−1^ dry weight) and the least in var. *lanceolata*, that recorded only 163.71 µg·g^−1^ dry weight (Table 3). Meanwhile, increasing the CO_2_ concentration from 400 to 1,200 µmol·mol^−1^ generally resulted in enhanced quercetin in all of the varieties. Under ambient CO_2_ levels the quercetin in var. *pumila* recorded the highest value at 105.66 µg·g^−1^ dry weight compared to var. *alata* and var. *pumila* that recorded 57.61 µg·g^−1^ dry weight and 56.90 µg·g^−1^ dry weight, respectively. With increasing CO_2_ concentration from 800 to 1,200 µmol·mol^−1^, the increase in quercetin concentration in var, *alata *(absolute) was by 3-fold (179%) and 4-fold (221%), var. *lanceolata* by about two and five fold (81%–260%); and lowest increases in quercetin occurred in var. *pumila* that recorded at only 10%–20% (Table 4). In the present study, elevated CO_2_ exposure did not enhance quercetin content as much in black tea and papaya that recorded 1,107 and 1,260 µg·g^−1^ dry weight values, respectively.

**Table 3:** The concentrations of flavonoid compounds in three varieties of *Labisia pumila* grown under different CO_2_ concentration.

CO_2_ levels (µmol·mol^−1^)	Varieties	Flavonoid content (µg·g^−1^ dry weight)				
		Kaempferol	Quercetin *	Myricetin	Rutin	Naringenin
	***Alata***	186.71 ± 0.34 ^b^	57.61 ± 1.22 ^g^	87.81 ± 0.34 ^c^	ND	139.20 ± 2.56 ^c^
**400**	***Pumila***	221.91 ± 0.21 ^a^	105.66 ± 2.11 ^f^	30.41 ± 2.33 ^e^	24.51 ± 0.45 ^c^	80.44 ± 0.98 ^d^
	***Lanceolata***	162.71 ± 0.31 ^c^	56.90 ± 2.34 ^g^	27.45 ± 3.11 ^f^	ND	87.11 ± 1.78 ^e^
	***Alata***	ND	160.88 ± 3.44 ^c^	273.84 ± 7.44 ^b^	ND	ND
**800**	***Pumila***	ND	117.42 ± 4.11 ^e^	ND	41.8 ± 3.22 ^b^	619.59 ± 9.78 ^b^
	***Lanceolata***	ND	103.13 ± 2.78 ^f^	49.73 ± 0.54 ^d^	ND	ND
	***Alata***	ND	183.32 ± 5.43 ^b^	287.77 ± 0.21 ^a^	ND	ND
**1200**	***Pumila***	ND	127.52 ± 0.45 ^d^	ND	87.45 ± 2.54 ^a^	947.85 ± 9.76 ^a^
	***Lanceolata***	ND	205.91 ± 0.21 ^a^	85.76 ± 1.45 ^c^	ND	ND

ND = not detected. All analyses are the mean of nine measurements ± standard error of mean. Results expressed in µg·g^−1^ of dry plant material. Means not sharing a common letter were significantly different at * p ≤ 0.05.

**Table 4:** Percent of increase or decrease of flavonoid compounds in three varieties of *Labisia pumila* grown under elevated CO_2_ concentration (800 and 1,200 µmol·mol^−1^)

Flavonoid compounds	Var *alata*		Var *pumila*		Var *lanceolata*	
	800 (µmol·mol^−1^)	1,200 (µmol·mol^−1^)	800 (µmol·mol^−1^)	1,200 (µmol·mol^−1^)	800 (µmol·mol^−1^)	1,200 (µmol·mol^−1^)
**Kaempferol**	−100%	−100%	−100%	−100%	−100%	−100%
**Quercetin**	+179%	+221%	+10%	+20%	+81%	+260%
**Myricetin**	+210%	+226%	−100%	−100%	+75%	+203%
**Rutin**	0%	0%	+70%	+262%	0%	0%
**Naringenin**	−100%	−100%	+683%	+1100%	−100%	−100%

Results expressed in percent; + and − indicate respectively increase and decrease of component concentrations when exposed to CO_2_.

The enhancement of myricetin concentration in CO_2_-enriched *L.pumila* was only observed in var. *alata* and var. *lanceolata*. Under 800 and 1,200 µmol·mol^−1^ CO_2_ enrichment myricetin in var. *alata* increased by 210% and 226%, respectively, compared to ambient conditions that just recorded 87.11 µg·g^−1^ dry weight (Table 3). In var. *lanceolata* myricetin concentration improved from 27.45 µg·g^−1^ dry weight in 400 µmol·mol^−1^ CO_2_ to 49.73 µg·g^−1^ dry weight in 800 µmol·mol^−1^ CO_2_ and 85.76 µg·g^−1^ dry weight under 1,200 µmol·mol^−1^ CO_2_ enrichment. Interestingly, myricetin disappeared in var. *pumila* when it was exposed to higher than ambient CO_2_. In contrast, the production of rutin and naringinin was only enhanced in var. *pumila* exposed to higher CO_2_ of 800 and 1,200 µmol·mol^−1^ CO_2_. There were no related increases found in the varieties *alata* and *lanceolata* with increasing CO_2_ level (Table 3). Under ambient conditions, rutin was only detected in var. *pumila*. As CO_2_ levels increased from 800 to 1,200 µmol·mol^−1^ CO_2_ rutin was enhanced by 70% and 260%, respectively in var. *pumila*. Meanwhile under ambient CO_2_ levels the naringenin content was highest in var. *alata*, recording values of 139.21 µg·g^−1^ dry weight, followed by var. *lanceolata *(87.11 µg·g^−1^ dry weight) and the lowest in var. *pumila *(80.44 µg·g^−1^ dry weight). Upon CO_2_ enrichment with 800 µmol·mol^−1^ CO_2_ and 1,200 µmol·mol^−1^ CO_2_ the naringenin content in var. *pumila* increased tremendously by 345% and 581%, respectively, compared to var. *alata* under ambient conditions. The naringenin content in var. *pumila* with a value of 947.85 µg·g^−1^ dry weight under 1,200 µmol·mol^−1^ CO_2_ was slightly low compared to grapefruit (1,150 µg·g^−1^ dry weight), that was found to contain the highest naringenin compared to other plant sources, but is relatively higher compared to tomato (448 µg·g^−1^ dry weight) and orange (887 µg·g^−1^ dry weight) [32,33]. Naringenin is a flavonoid that is considered to have a bioactive effect on human health as an antioxidant, free radical scavenger, anti-inflammatory, carbohydrate metabolism promoter, and immune system modulator [20]. The increase in quercetin and naringenin in the present study under elevated CO_2_ have also been documented by Ghasemzadeh *et al*. [20] in ginger where they observed increased quercetin and naringenin to be about 26% and 480%, respectively, in ginger leaves when exposed to 800 µmol·mol^−1^ CO_2_. 

The increase in production of total phenolics and flavonoids in the present study might be due to increased production of total non structural carbohydrates when plants are exposed to elevated CO_2_. The same observation was also reported by Wang *et al.* [34] and Malikov *et al.* [35] in *Fragaria ananassa* and *Betula pendula*, respectively, when exposed to high CO_2_ levels. Ibrahim *et al. *[19] previously have indicated that the increased production of secondary metabolites in *L. pumila* under elevated CO_2_ was due to an accumulation of starch and soluble sugars under high exposure to CO_2_ (800 and 1,200 µmol·mol^−1^). In other study, Ibrahim and Jaafar [36] also found that the increase in the production of total phenolics and flavonoids was concomitantly followed by an increase in the production of fructose. The increase in production of secondary metabolites by the increase in fructose content had been observed by Hilal *et al*. [37] in quinoa seedlings where the increase in total phenolics and flavonoids of the seedlings under UV-B radiation was significantly related to an increase in fructose content in quinoa leaves. The up-regulation of plant secondary metabolites with increasing fructose levels under elevated CO_2_ might be due to enhanced activity of the pentose phosphate pathway to supply high levels of erythose-4-phosphate, which is used as a substrate for the synthesis of lignin and secondary metabolite compounds in the shikimic acid pathway [38]. The increase in the production of erythose-4-phoshate implies a high production of fructose because both these compounds are synthesized in the same reaction in the pentose phosphate pathway catalyzed by transaldolase [39]. The current results suggest that the increased production of secondary metabolites in *L. pumila* under elevated CO_2_ might be due to enhanced production of erythose-4-phosphate under this condition. 

Wang *et al*. [34], obtained the same result when CO_2_ was enriched at 950 µmol·mol^−1^ in strawberry the concentrations of flavonoid compounds such as *p*-coumaroylglucose, dihydroflavonol, quercetin 3-glucoside, quercetin 3-glucuronide, kaempferol 3-glucoside contents, cyanidin 3-glucoside, pelargonidin 3-glucoside, and pelargonidin 3-glucoside succinate were significantly enhanced. The same finding was also observed with Chabot *et al.* [40] who showed the ability of elevated CO_2_ concentrations to enhance flavonoid components in *Gigaspora margarita* where the exposure to high CO_2_ enhanced flavonoid compounds like quercetin, morin, hesperetin, genistein and chrysin. Estiarte *et al.* [41] also found exposure that exposure of *Triticum aestivum* c.v. Yecora Rojo to elevated CO_2_ at 550 µmol·mol^−1^ enhanced the production of flavonoids and their components. They observed that a flavonoid compound (isoorientin) content was enhanced by 14% under elevated CO_2_ conditions. The interspecific difference in the phenolics and flavonoid compounds in this study might be due to the differences in enyzme stimulation for each compound, precursor availability, differing biosynthetic pathways, genetic and carbon allocation to plant parts either for storage or structural components.

Stimulation of secondary metabolite production of *L. pumila* under elevated CO_2_ in the present study might be due to increased availability of carbon uptake due to enhancement of leaf gas exchange properties [27]. Under elevated CO_2_ net photosynthesis was up-regulated, the increase in net photosynthesis with high levels of CO_2_ increased the production of total non-structural carbohydrates. The increase in production of total non-structural carbohydrate under elevated CO_2_ was observed in *Citrus aurantrium* when exposed to 670 µmol·mol^−1^ CO_2_ [42]. Plant polyphenols (flavonoid and phenolics) are biosynthesized via several routes and thus constitute a heterogeneous group from the metabolic point of view. The two basic pathways involved are the shikimic acid and the malonic acid pathways. The shikimic acid pathway is able to convert simple carbohydrate precursors derived from glycolysis and pentose phosphate pathway into aromatic amino acids [20]. Previous studies have shown that the increase in phenolic and flavonoid compound is related to the balance between carbohydrate sources and sinks, such that greater source or sink ratio under exposure to elevated CO_2_ will results in higher phenolic concentration and compounds [43,44]. The present result suggest that enhancement of leaf gas exchange under elevated (photosynthesis) have increase the availability of carbohydrate that enhance the production of phenolics and flavonoid content in *L. pumila*. 

### 2.3. Phenylalanine Ammonia Lyase (PAL) Activity

**Figure 3:**
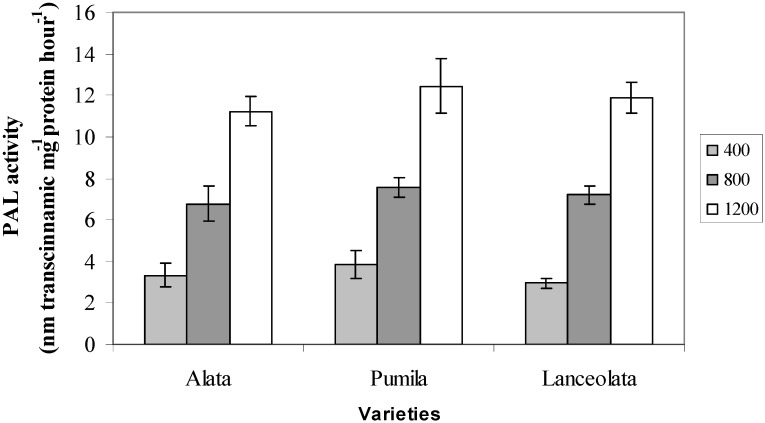
The effects of different CO_2_ concentration on PAL activity of three varieties of *L. pumila*. N = 9. Bars represent standard error of differences between means (SEM).

The PAL activity was influenced by CO_2_ concentrations (*p* ≤ 0.05; Figure 3). In all the three varieties PAL activity was found to be consistently highest under 1,200 µmol^−1^·mol^−1^ CO_2_ with values ranging between (11.24–12.45 nM transcinnamic mg^−1^ protein hour^−1^) than at 800 µmol^−1^·mol^−1^ CO_2_ that recorded PAL activity just 6.78 to 7.56 nM transcinnamic mg^−1^ protein hour^−1^. Under ambient CO_2_ conditions, PAL activity in the three varieties registered the lowest values, between 2.94 and 3.87 nM transcinnamic mg^−1^protein hour^−1^. The present study showed that as CO_2_ levels increased, the production of PAL activity was also enhanced. It can be hypothesized that the increase in production of secondary metabolites in the present work could be attributed to an increase in PAL activities under high CO_2_ levels. This is basically due to the fact that PAL is a precursor to total phenolics and flavonoids biosynthesis [45,46]. The increase in PAL activity in the current study might also be related to reduction in nitrogen content in plant exposed to high CO_2_ levels which could have increased the availability of phenylalanine (phe) due to restriction in the protein production that enhanced phenylalanine partitioning to the production of CBSM [47]. The increase in PAL activity was usually followed by increase in C/N ratio due to enhanced growth rate under elevated CO_2_ that easily reduced nitrogen content. Winger *et al.* [48] indicated that increases in the C/N ratio in plants were an indication of increases in the synthesis of plant secondary metabolites, especially phenolics and flavonoids. The increase in PAL activity under high CO_2_ was also observed by Matros *et al.* [49] and Hartley *et al.* [50] in tobacco and *Spergula avensis*, respectively. 

### 2.4. Radical Scavenging Activity (DPPH)

**Figure 4:**
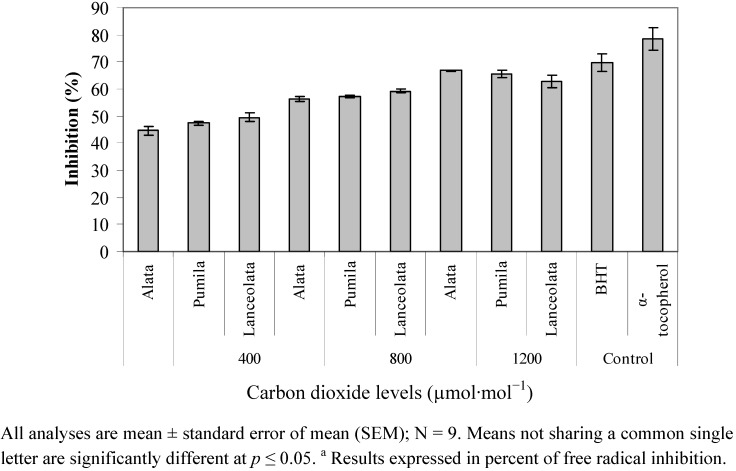
Leaf DPPH scavenging activities in different varieties of *L. pumila* under different CO_2_ levels. BHT and α-tocopherol were used as positive controls.

Generally, the highest DPPH antioxidant activity was recorded with 1,200 µmol·mol^−1^ CO_2_ followed by 800 µmol·mol^−1^ CO_2_ and the lowest for 400 µmol·mol^−1^ CO_2_ treatment. The treatment effects of DPPH were contributed by CO_2_ levels (*p* ≤ 0.05; Figure 4). At 370 µg/mL, the DPPH antioxidant activity recorded the highest inhibition value (62.89–66.74%) when exposed to 1,200 µmol·mol^−1^CO_2_, followed by the 800 µmol·mol^−1^ CO_2_ (56.21–59.24%), and the least in the 400 µmol·mol^−1^ CO_2_ treatment (44.62–49.52%). This study showed that *L. pumila* methanolic extract has a good free radical scavenging activity, and, hence, it can be used as a radical scavenging agent acting possibly as a primary antioxidant. This result also implies that exposure to high CO_2_ concentrations could signiﬁcantly enhance the DPPH radical scavenging activity of a medicinal plant. It is noteworthy that the DPPH assay principally measures the activity of water-soluble antioxidants [51]. Results of the current work suggest that high CO_2_ supply is advantageous to *L. pumila* in the improvement of the antioxidant activity of water-soluble antioxidants. In our previous study, besides phenolics and flavonoid compounds, other water-soluble antioxidants of the extracts such as ascorbic acid and anthocyanin could also exert an additive effect on DPPH radical scavenging activity. 

### 2.5. Reducing Ability (FRAP)

**Figure 5:**
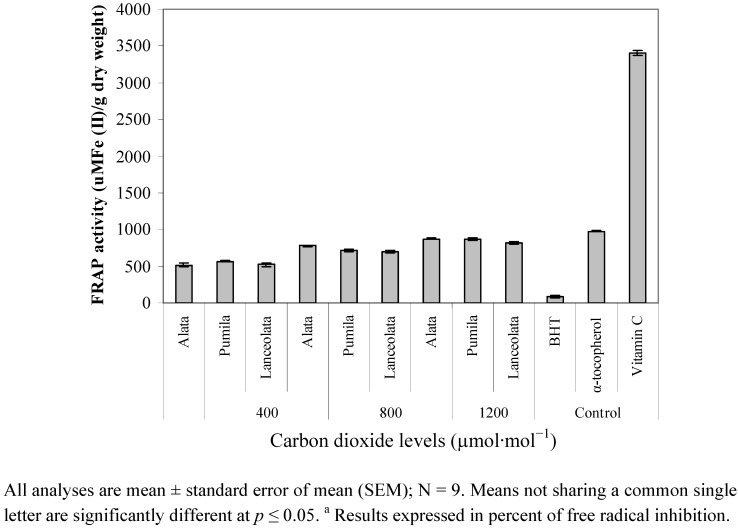
Leaf total antioxidant activity (FRAP) in different varieties of *L. pumila* under different CO_2_ levels. BHT and α-tocopherol were used as positive controls.

The ferric reducing antioxidant potential (FRAP) was influenced by CO_2_ exposure (*p* ≤ 0.01). The FRAP assay is very simple, fast and precise, and was recently developed to measure the total antioxidant power of biological ﬂuids [52]. The FRAP activity was found to followed similar trend with DPPH antioxidant activity where the highest activity was observed in 1,200 µmol·mol^−1^ CO_2_, followed by 800 µmol·mol^−1^ CO_2_ and 400 µmol·mol^−1^ CO_2_ (Figure 5). The reducing ability of extracts from different varieties of *L. pumila* with 1,200 µmol·mol^−1^ CO_2_ was in the range of 817.14 to 874.26 µm of Fe(II) dry weight, whilst at 800 µmol·mol^−1^ CO_2_ the improvement was only 700.27 to 777.24 µm Fe(II) dry weight. Comparatively, at ambient CO_2_ concentration of 400 µmol·mol^−1^ CO_2_ a low reducing ability of the leaf extract (516.33 to 570.12 µm of Fe(II) dry weight; Table 5) was observed. The FRAP values for the methanolic extracts of the leaves, were statistically and significantly lower than vitamin C and α-tocopherol, but higher than that of BHT. Our finding is in agreement with that of Wang *et al.* [34], who reported the increase in FRAP activity in strawberry fruit by 24% as exposed to elevated CO_2_ concentrations at 950 µmol·mol^−1^CO_2_.The enhancement of DPPH and FRAP activity under elevated CO_2_ in the present study might be due to increase in production of total phenolics and flavonoids under high levels of CO_2_. The present study suggest that the increase antioxidant capabilities under elevated CO_2_ might be to up-regulation of production of plant secondary metabolites that were characterized high production of phenolics and flavonoids compounds.

## 3. Experimental

### 3.1. Experimental Location, Plant Materials and CO_2_ Treatments

The experiment was carried out under a growth house at Field 2, Faculty of Agriculture Glasshouse Complex, Universiti Putra Malaysia (longitude 101°44'N and latitude 2°58'S, 68 m above sea level) with a mean atmospheric pressure of 1.013 kPa. Three-month old *L. pumila* seedlings that were propagated from leaf cuttings of var. *alata*, var. *pumila* and var. *lanceolata* were left for a month in a nursery to acclimatize until they were ready for the treatments. The seedlings were planted in soil-less medium containing coco-peat, burnt paddy husk and well composted chicken manure in 5:5:1 (v/v) ratio in 25-cm diameter polyethylene bags. Organic fertilizer was applied every two weeks using well composted chicken manure at 20 g/plant. Carbon dioxide enrichment treatment started when the seedlings reached four months of age where plants were exposed to 400, 800 and 1,200 µmol·mol^−1^ CO_2_. This 2-factorial experiment was arranged in a split plot using a randomized complete block design with CO_2_ levels being the main plot, and varieties as the sub-plot replicated three times. Each treatment consisted of nine seedlings. The seedlings were raised in specially constructed growth houses receiving 12-h photoperiod and average photosynthetic photon flux density of 300 µmol·m^−2^s^−1^. Day and night temperatures were recorded at 30 ± 1.0 °C and 20 ± 1.5 °C, respectively, and relative humidity at about 70% to 80%. Vapor pressure deficit ranged from 1.01 to 2.52 kPa. Carbon dioxide at 99.8% purity was supplied from a high-pressure CO_2_ cylinder and injected through a pressure regulator into fully sealed 2 m × 3 m growth houses at 2-h daily and applied continuous from 08:00 to 10:00 a.m. The CO_2_ distributed inside the growth house by having two opening at the edge of the constructed growth chamber that were mounted a small fan. During enrichment, small low r.p.m. fans were simultaneously used to disperse CO_2_ evenly inside the constructed growth house. The CO_2_ concentration at different treatments was measured using Air Sense™ CO_2_ sensors designated to each chamber during CO_2_ exposition period. Plants were watered three to four times a day at 5 min per session to ensure normal growth of plant using drip irrigation with emitter capacity of 2 L·h^−1^. The experiment lasted for 15 weeks from the onset of treatment [53,54].

### 3.2. Preparation of Extracts for RP-HPLC

Samples were extracted using methanol as a solvent as described by Crozier *et al.* [55]. Two grams of freeze-dried leaf were weighed and placed into a 100 mL conical flask, and treated with 80% (v/v) aqueous methanol (40 mL). It was followed by an addition of 6 M HCl (10 mL). The mixture was refluxed for 2 h at 90 °C and filtered using Whatman No. 1 filter paper (Whatman, Durham, UK) followed by evaporation of the filtrate using a vacuum rotary evaporator (Buchi, Arbon, Switzerland). The crude extracts were re-dissolved in methanol for RP-HPLC analyses.

### 3.3. Analyses of Phenolic and Flavonoid Compounds by RP-HPLC

The phenolic and flavonoid compounds of the leaf of *Labisa pumila* were quantitatively measured by reversed-phase HPLC based on the method described by Crozier *et al.* [55] with some modifications. Phenolic standards used were gallic acid, caffeic acid and pyrogallol. Flavonoid standards were quercetin, rutin, myricetin, kaempferol and naringin at stock concentrations of 100 µg/mL. An aliquot of sample extract was loaded on the HPLC equipped with an Intersil ODS-3 (5 μm 4.6 × 150 mm, Gl Science Inc) analytical column. Solvents comprising deionised water (solvent A) and acetonitrile (solvent B) were used. The pH of water was adjusted to 2.5 with trifluoroacetic acid. The phenolic and isoflavonoid compounds were detected at 280 nm while flavonoid compounds at 350 nm. The column was equilibrated by 85% solvent A and 15% solvent B. Then the ratio of solvent A was increased to 85% in 50 min followed by reducing solvent B to 15% in 55 min. This ratio was maintained to 60 min for the next analysis with flow rate at 0.6 mL/min.

### 3.4. Phenylalanine-Ammonia-Lyase (PAL)

Phenylalanine-ammonia-lyase (PAL) activity was measured using the method described by Martinez and Lafuente [56]. The enzyme activity was determined by measuring spectrophotometrically the production of *trans*-cinnamic acid from L-phenylalanine. Enzyme extract (10 µL) was incubated at 40 °C with 12.1 mM L-phenylalanine (90 µL, Sigma, Chicago, IL, USA) that were prepared in 50 mM Tris-HCl, (pH 8.5). After 15 min of reaction, *trans*-cinnamic acid yield was estimated by measuring increase in the absorbance at 290 nm. Standard curve was prepared by using a *trans*-cinnamic acid standard (Sigma) and the PAL activity was expressed as nM *trans*-cinnamic acid µg^−1^ protein h^−1^.

### 3.5. DPPH Radical Scavenging Assay

The DPPH free radical scavenging activity of each sample was determined according to the method described by Ibrahim *et al.* [57]. A solution of 0.1 mM DPPH in methanol was prepared. The initial absorbance of the DPPH in methanol was measured at 515 nm. An aliquot (40 µL) of an extract was added to 3 mL of methanolic DPPH solution. The change in absorbance at 515 nm was measured after 30 min. The antiradical activity (AA) was determined using the following formula: 


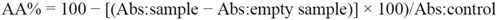


The optic density of the samples, the control and the empty samples were measured in comparison with methanol. One synthetic antioxidant, BHT (butylated hydroxytoluene) and α-tocopherol, were used as positive controls. The antioxidant capacity based on the DPPH free radical scavenging ability of the extract was expressed in percent of free radical inhibition 

### 3.6. Reducing Ability (FRAP Assay)

The ability to reduce ferric ions was measured using modifying methods of Benzie and Strain [52]. An aliquot (200 µL) of the extract with appropriate dilution was added to FRAP reagent (3 mL, 10 parts of 300 mM sodium acetate buffer at pH 3.6, 1 part of 10 mM TPTZ solution and 1 part of 20 mM FeCl_3_·6H_2_O solution) and the reaction mixture was incubated in a water bath at 37 °C. The increase in absorbance at 593 nm was measured after 30 min. The antioxidant capacity based on the ability to reduce ferric ions of the extract was expressed as expressed in µM Fe(II)/g dry mass and compared with those of standards for BHT, ascorbic acid, and α-tocopherol.

### 3.7. Statistical Analysis

Data were analyzed using analysis of variance by SAS version 17. Mean separation test between treatments was performed using Duncan multiple range test and standard error of differences between means was calculated with the assumption that data were normally distributed and equally replicated [58,59].

## 4. Conclusions

The manipulation of CO_2_ may be an effective method to increase the expression of phenolic and flavonoid compounds in *L. pumila*. The phenolic and flavonoid profiles are dependent on the interspecific variability between CO_2_ levels and *L. pumila* varieties. High performance liquid chromatography (HPLC) results showed that gallic acid and quercetin were the most abundant phenolic and flavonoid acids in Malaysian *L. pumila* varieties, which can be further improved with elevated CO_2_. From the study, it was noted that the increase in the production of phenolics and flavonoids under elevated CO_2_ might be due to enhanced PAL activity as CO_2_ levels increased. From the study, it was observed that the increase in production of plant secondary metabolites in *L. pumila* was followed by enhancement of the antioxidant activity (DPPH and FRAP) under exposure of elevated CO_2_. The present results showed the potential use of CO_2_ enrichment techniques for enhancement of medicinal properties of this plant for future cultivation.
